# Telehealth strategies in the care of people with chronic kidney disease: integrative review

**DOI:** 10.1590/1518-8345.6824.4050

**Published:** 2023-12-04

**Authors:** Onislene Alves Evangelista de Almeida, Maria Eduarda Freitas de Lima, Walterlânia Silva Santos, Bárbara Louise Moreira Silva

**Affiliations:** 1 Universidade de Brasília, Brasília, DF, Brasil; 2 Hospital Universitário de Brasília, Centro de Diálise, Brasília, DF, Brasil; 3 Universidade de Brasília, Faculdade de Ceilândia, Brasília, DF, Brasil

**Keywords:** Chronic Kidney Disease, Dialysis, Peritoneal Dialysis, Kidney Transplant, Telenursing, Telehealth, Enfermedad Renal Crónica, Diálisis, Diálisis Peritoneal, Trasplante de Riñón, Teleenfermería, Telesalud, Falência Renal Crônica, Diálise, Diálise Peritoneal, Transplante de Rim, Telenfermagem, Telemedicina

## Abstract

**Objective:**

to evaluate the evidence about telehealth strategies in caring for people with chronic kidney disease.

**Method:**

integrative literature review. The search for primary studies was carried out in six databases: PubMed/MEDLINE, Web of Science, EMBASE, CINAHL, LILACS, and Scopus. The sample consisted of 48 articles published between 2000 and 2021. The telehealth strategy was applied by a multidisciplinary team of doctors, nurses, pharmacists, nutritionis, and social workers. The type of study, country, strategy applied, setting, population, and professional were extracted from the articles. The studies were selected by reading the title and abstract (phase 1) and then reading them in full (phase 2), categorizing them by telehealth strategy. The results were summarized descriptively and the studies were classified according to their level of evidence.

**Results:**

the home was the most representative in dialysis and conservative treatment. Six categories of telehealth strategies were identified: remote monitoring devices, teleconsultation, digital platforms, apps, multimodality strategies, and telephone contact.

**Conclusion:**

using these strategies for the care of people with chronic kidney disease presents different forms and implementations, being feasible for the renal population at any stage of the disease and applicable by different health professionals with an emphasis on the home environment. The evidence shows that telehealth favors lower cost, accessibility to remote locations, and better monitoring of dialysis with positive resul in symptom control, risk reduction, and patient training.

## Abstract (es)

**Objetivo:**

evaluar las evidencias sobre estrategias de telesalud en la atención de personas con enfermedad renal crónica.

**Método:**

revisión integradora de la literatura. La búsqueda de estudios primarios se realizó en seis bases de datos: PubMed/MEDLINE, *Web of Science*, EMBASE, CINAHL, LILACS y Scopus. La muestra estuvo compuesta por 48 artículos publicados entre 2000 y 2021, la estrategia de telesalud fue aplicada mediante equipo multidisciplinario, médico, enfermero, farmacéutico, nutricionista y trabajador social. De los artículos se extrajo el tipo de estudio, país, estrategia aplicada, escenario, población y profesional. Los estudios fueron seleccionados mediante la lectura de título y resumen (fase 1) y, posteriormente, mediante la lectura completa (fase 2), se los categorizó por estrategia de telesalud. El resumen de resultados se presentó de forma descriptiva y los estudios se clasificaron según el nivel de evidencia.

**Resultados:**

el domicilio fue el más representativo en las terapias de diálisis y tratamiento conservador. Se identificaron seis categorías de estrategias de telesalud: dispositivos de monitoreo remoto, teleconsulta, plataforma digital, aplicaciones, estrategias multimodales y contacto telefónico.

**Conclusión:**

el uso de esas estrategias para la atención de personas con enfermedad renal crónica tiene diferentes formatos e implementaciones, las mismas son factibles para dicha población en cualquier etapa de la enfermedad y pueden ser aplicadas por diferentes profesionales de la salud especialmente en el ámbito domiciliario. La evidencia ha demostrado que la telesalud favorece la reducción de costos, la accesibilidad a ubicaciones remotas, un mejor seguimiento de la diálisis con resultados positivos sobre el control de los síntomas, la reducción de riesgos y capacitación del paciente.

## Abstract (pt)

**Objetivo:**

analisar as evidências sobre as estratégias de telessaúde no atendimento às pessoas com doença renal crônica.

**Método:**

revisão integrativa da literatura. A busca pelos estudos primários foi realizada em seis bases de dados: PubMed/MEDLINE, Web of Science, EMBASE, CINAHL, LILACS e Scopus. A amostra foi composta por 48 artigos publicados entre 2000 e 2021, a estratégia de telessaúde foi aplicada por equipe multidisciplinar, médico, enfermeiro, farmacêutico, nutricionista e assistente social. Foram extraídos dos artigos o tipo de estudo, país, estratégia aplicada, cenário, população e profissional. Os estudos foram selecionados por leitura de título e resumo (fase 1) e, após, por leitura completa (fase 2), com sua categorização por estratégia de telessaúde. A síntese dos resultados foi apresentada de forma descritiva e os estudos classificados de acordo com o nível de evidência.

**Resultados:**

o domicílio foi o de maior representatividade nas terapias dialíticas e tratamento conservador. Foram identificadas seis categorias de estratégias de telessaúde: dispositivos de monitoramento remoto, teleconsulta, plataforma digital, aplicativos, estratégias multimodalidades e contato telefônico.

**Conclusão:**

a utilização dessas estratégias para o atendimento de pessoas com doença renal crônica apresenta diferentes formatos e implementações, sendo viável à população renal em quaisquer fases da doença e aplicável por diferentes profissionais de saúde com ênfase no ambiente domiciliar. As evidências apontaram que a telessaúde favorece a diminuição de custos, acessibilidade aos locais afastados, melhor monitoramento da diálise com resultados positivos no controle dos sintomas, redução dos riscos e treinamento do paciente.

**Highlights:**

**(1)** Telehealth in chronic kidney disease care is feasible and promising.

**(2)** Telehealth is feasible for people at all stages of CKD.

**(3)** Health promotion and monitoring were the most applied by telehealth.

**(4)** Remote care can reduce costs, emergencies, and contac with the clinic.

**(5)** Nurses mainly used telephone contact and teleconferencing.

## Introduction

Chronic kidney disease (CKD) is a complex public health problem and affects around 8% to 16% of the world’s population, as well as being associated with high cardiovascular risk and death ^(^ 1 ^)^. It is characterized by slow, progressive, and irreversible changes in kidney function or structure, the main causes of which are arterial hypertension, Diabetes Mellitus, and glomerulonephritis, among others ^(^ 2 ^)^. According to its progression - evidenced by the drop in glomerular filtration rate - CKD is classified into five stages that require specific treatment and monitoring depending on the degree of kidney damage. The fifth and final stage is marked by the terminal phase of chronic renal failure, in which Renal Replacement Therapies (RRT) such as Hemodialysis (HD), Peritoneal Dialysis (PD), and/or transplantation are necessary ^(^ 1 ^-^ 2 ^)^. 

A multi-professional approach is essential in the optimized management of CKD, in terms of dietary control, pharmacotherapy, adjusting risk factors, and promoting self-care. Given its complexity, health professionals must be aligned with the tools available for effective interventions in the care of these patients, including the possibilities offered by digital technology ^(^ 3 ^)^. 

In this sense, Telehealth (TH), which covers the most diverse categories of remote care, presents itself as a viable and safe strategy to support the needs of chronic kidney patients ^(^ 4 ^-^ 5 ^)^. TH involves providing and promoting long-distance clinical care, health education for patients and professionals, public health, and health administration ^(^ 5 ^-^ 6 ^)^. Thus, it is understood that apps of telehealth include: digital media, short message service, mobile apps, interactive voice response, videoconferencing, asynchronous storage communication, routing, and wireless communication ^(^ 3 ^,^ 6 ^)^. 

Despite the expansion of telehealth during the COVID-19 pandemic, successful experiences before this period have shown the positive impact of telemedicine in the care of chronic conditions, such as Diabetes Mellitus ^(^ 7 ^,^ 8 ^)^. These results demonstrate the potential for the app of telemedicine in CKD, in the sense of leveraging multidisciplinary care in the area, breaking with traditional care methodologies, and fostering self-responsibility in those who live with this condition daily ^(^ 5 ^)^. This could be strongly experienced during the pandemic when TH was widely used to maintain and provide care to users and comply with the rules restricting the movement of people needed to combat the pandemic. 

However, the application of TH in clinical practice in Nephrology, although it has expanded, still presents obstacles and needs to be discussed from various aspects - clinical, ethical and normative ^(^ 10 ^)^. In clinical practice, it is important to assess the effectiveness and cost-benefit of these technological practices compared to traditional care, as well as determine which technologies and how it is being applied by the various professionals in the multidisciplinary team who provide care to people with CKD. The path taken by TH in the field of Nephrology continues to expand, but it is essential to understand this process and how its players behave in an environment of atypical and increasingly digital resources ^(^ 3 ^)^. 

Thus, telehealth in Nephrology has diverse approaches, in different contexts of CKD care and by any health professional ^(^ 5 ^)^ indicating the need to synthesize the evidence published in the area. Thus, this review aims to evaluate the evidence about telehealth strategies in caring for people with CKD. 

## Method

### Type of study

This is an integrative review study carried out in six stages: definition of the research problem, design of inclusion and exclusion criteria for the studies, categorization of those included, evaluation, interpretation, and final presentation ^(^ 11 ^)^. The protocol for this review can be made available on request to the authors. The publication selection process followed the recommendations of the Preferred Reporting Items for Systematic Reviews and Meta-Analyses (PRISMA) ^(^ 12 ^)^. 

### Place and period

This study was carried out in the city of Brasília/Brazil and took place between February 2021 and December 2022. The selection of articles by title and abstract took place between March and April, and the selection by a full reading of potential studies between May and September. The information was analyzed between October 2021 and February 2022.

### Delimitation of the sample

The research problem - structured by the acronym PICO (P= people with CKD; I= telehealth; C= not applicable; O= clinical, laboratory and behavioral) - was: what evidence is available in the literature on telehealth strategies in the care of people with CKD? The controlled descriptors - Chronic Renal Failure, Dialysis, Transplantation and Telemedicine - were combined with the Boolean operators *AND* and *OR* in the PubMed/MEDLINE, Web of Science, EMBASE, Cumulative Index to Nursing and Allied Health Literature (CINAHL), Latin American and Caribbean Health Sciences Literature (LILACS) and Scopus databases through the journal portal of the Coordination for the Improvement of Higher Education Personnel (CAPES), in March 2021. 

The strategy applied to the PubMed and Scopus databases was created using the *Medical Subject Headings* (MeSH) in the following format: ( *“Kidney Failure, Chronic”[Mesh] OR “Kidney Failure, Chronic” OR “End-Stage Kidney Disease” OR “Disease, End- Stage Kidney” OR “End-Stage Kidney Disease” OR “End-Stage Renal Disease” OR “Chronic Renal Insufficiency” OR “End-Stage Renal Disease” OR “End-Stage Renal Disease” OR “End-Stage Renal Disease” OR “End-Stage Renal Disease” OR “End-Stage Renal Disease” OR “End-Stage Renal Disease” OR “End-Stage Renal Insufficiency” OR “End-Stage Renal Insufficiency” OR “End-Stage Renal Insufficiency” OR “Chronic Renal Insuff, Chronic” OR “Chronic Renal Failure” OR “ESRD” OR “Renal Failure, Chronic” OR “Renal Failure, Chronic” OR “Renal Failure, Chronic” OR “Renal Failure, Chronic” OR “Renal Failure, Chronic” OR “Renal Failure, Chronic” OR “Kidney Diseases, Chronic” OR “Chronic Renal Diseases, Chronic” OR “Kidney Diseases, Chronic” OR “Kidney Diseases, Chronic” OR “Kidney Diseases, Chronic” OR “Kidney Diseases, Chronic” OR “Kidney Diseases, Chronic” OR “Chronic Renal Diseases, Chronic” OR “Kidney Diseases, Chronic” OR “Chronic Renal Diseases, Chronic” OR “Chronic Renal Disease” OR “Disease, Chronic Renal” OR “Diseases, Chronic Renal” OR “Renal Disease, Chronic Renal” OR “Renal Disease, Chronic” OR “Renal Diseases, Chronic”) AND (“Telemedicine Emergency Care” OR “Telemedicine” OR “Mobile Health” OR “Health, Mobile” OR “mHealth” OR “Telehealth” OR “eHealth” OR “Telemonitoring” OR “Teletherapy” OR “Telescreening, Medical”).* Similar search strategies were adopted for the other databases, with specific vocabularies according to the database, such as CINAHL Headings, Health Sciences Descriptors (DeCS), and Entry Terms for CINAHL, LILACS, and Embase, respectively. 

The EndNote Web Basic (Clarivate Analytics ^®^) software was used to identify and extract duplicates, and the Rayyan Qatar Computing Research Institute (Rayyan QCRI) platform ^(^ 13 ^)^ was used to select the studies in phase 1. 

### Selection criteria

The inclusion criteria were: interventions with telehealth, previously structured and applied by health professionals in adult patien with CKD - whether they are undergoing conservative treatment, dialysis, or transplantation, observational research - quasi-experimental, Clinical Trials (CT) with full text available in English, Spanish or Portuguese, with no restrictions on the period of publication. Consequently, the exclusion criteria were defined as research on the interest and usability of technologies, reviews, case studies, expert opinions, research protocols, hospital context, and different publications with the same intervention and sample.

### Variables and data analysis

Two independent reviewers carried out the selection by title and abstract (phase 1) and the complete reading of the studies listed in the first selection (phase 2), followed by the extraction of data from those considered to be included. The same eligibility criteria were applied in both selection phases. An electronic spreadsheet (Microsoft Excel 2010 ^®^) was used to gather the information of interest: authors, title, year of publication, study objective, country, methodological design, setting and population, telehealth strategy, results, and limitations reported. 

The level of evidence was assessed according to the following classification: Level I (meta-analyses of randomized studies), Level II (experimental), Level III (quasi-experimental), Level IV (observational, cohort, case-control), Level V (systematic reviews of observational studies and qualitative studies), Level VI (single descriptive or qualitative study) and Level VII (opinions), with Levels I to IV being considered strong to moderate evidence ^(^ 14 ^)^. The included studies were organized categorically according to the telehealth strategies applied while the intervention and the information extracted were synthesized descriptively and presented in a summary table. 

## Results

**Figure 1 -:**
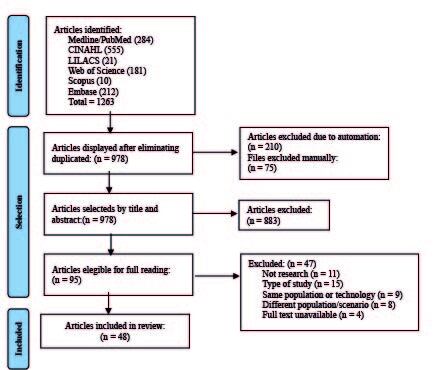
Flowchart of the selection process for the studies included in the integrative review according to the Preferred Reporting Items for Systematic Review and Meta-Analyses (PRISMA). Brasília, DF, Brazil, 2021

The identification of potential studies through the databases retrieved 1,263 articles, of which 285 were duplicates. After selection by title and abstract based on the inclusion and exclusion criteria, 95 articles remained, which were read in full in a second stage that resulted in the inclusion of 48 publications in this review. This identification and selection process is illustrated in Figure 1. 

Given the focus on telehealth strategies identified in the selected studies, the descriptive analysis resulted in six thematic categories: remote monitoring devices, teleconsultation, digital platforms, apps, multimodality strategies and telephone contact. Figure 2 brings together the main data extracted from the articles included by thematic category. 

**Figure 2 -:** Description of the characteristics of the studies included in the integrative review (n=48). Brasília, DF, Brazil, 2021

Authors (year)/ Periodical/ Country	Delineation / Scenario/ Population	Objective	Strategies	Results / Level of Evidence
**1. Remote Monitoring Devices**				
Li, et al., 2020 / Journal of Medical Internet Research/ Taiwan	RCT ^ * ^ Home-based/CKD ^ † ^ stages 1 to 4	To evaluate the effectiveness of the health management and social media platform in improving self-management skills and delaying the progression of ^ † ^ CKD.	MR ^ ‡ ^ by device (wristwatch) and social media platform.	IG ^ § ^ had higher scores for self-efficacy, self-management, quality of life, and increased number of steps *per* day, with a smaller decline in GFR ^ || ^ ./ Level II
St-Jules, et al., 2020/ Journal of Renal Nutrition/ United States	RTC ^ * ^ Home-based / HD ^ ¶ ^ with hyperphosphatemia	Examine the feasibility and acceptability of the self-directed mobile health program with education, self-monitoring, and behavioral counseling on phosphorus control.	Educational programs through videos and handou associated with self-monitoring with feedback by e-mail.	Nurse-supervised RM ^ ‡ ^ can improve health outcomes for patients with high-risk CKD ^ † ^ by reducing hospitalizations and emergency room visits./ Level II
McGillicuddy, et al., 2020/ Annals of Pharmacotherapy/ United States	RTC ^ * ^ / Home-based/ Post-transplanted kidneys	To determine whether mHealth affects intra-patient variability in tracolimus adherence.	Electronic medicine tray integrated into the app Smartphone Medication Adherence Saves Kidneys (SMASK).	IG ^ § ^ achieved a significant reduction in mean tacrolimus at 12 months (p = 0.046) and a significant improvement in the proportion achieving the low tacrolimus coefficient of variation (<40%; p = 0.001), compared to GC ^ ** ^ ./ Level II
Manani, et al., 2020/ Journal of Nephrology/ Italy	Retrospective case-control/ Home-based / PD ^ †† ^	To compare clinical outcomes and QoL ^ ‡‡ ^ between patients using or not using MR ^ ‡ ^ through PD ^ †† ^ machines.	Claria Cycling Machine ^®^.	Statistically significant reduction in the number of hospitalizations for specific diseases, emergencies, and acute hypervolemia in the RM ^ ‡ ^ group. There were no differences between the two groups in terms of all-cause hospitalization, hypervolemia, and QoL ^ ‡‡ ^ ./ Level IV
Viglino, et al., 2019/ Journal of Nephrology/ Italy	Transverse/ Home-based / PD ^ †† ^	Describe the telemedicine system created to overcome the physical, cognitive, and psychological barriers to PD ^ †† ^ .	The video dialysis system consists of a remote station in the patient’s home and a station in the Control Center equipped with a video camera, monitor, microphone, technological connectivity box, high-resolution computer, webcam, and speakerphone.	There were no differences related to peritonitis in the groups evaluated. Video-assisted PD ^ †† ^ proved to be highly reliable and easy to use for staff, patients, and caregivers, without requiring special technological skills.
Sanabria, et al., 2019/ Peritoneal Dialysis International/ Colombia	Retrospective cohort/ Home-based/ PD ^ †† ^	To evaluate the association between the use of RM ^ ‡ ^ , hospitalizations, and hospital days.	Claria Cycling Machine ^®^.	Significant reduction in the hospitalization rate in the RM ^ ‡ ^ group compared to the non-monitored group./ Level IV
Ellis, et al., 2019/ JMIR Formative Research/ United States	Prospective cohort/ Home-based / CKD ^ † ^ stage 1 to 4	To evaluate the feasibility and acceptability of using the mobile health system for medication adherence.	mHealth system with a smart button device for tracking medication intake through the smartphone app and SMS ^ §§ ^ service.	Of the 260 expected data points, 36.5% were recorded with the smart button and 76.2% with electronic monitoring. Sending encouraging text messages and medication schedule reminders were suggested./ Level IV
Magnus, et al., 2017/ Applied Clinical Informatics/ United States	Transverse/ Home-based / PD ^ †† ^	Describe satisfaction with the telehealth interface and health outcomes associated with the intervention.	Telehealth monitoring with RM ^ ‡ ^ for blood pressure, weight, and glucose, video chat, and access to online educational videos.	RM ^ ‡ ^ was associated with the perception of autonomy and confidence in health activities. There was a decrease in negative perceptions of PD ^ †† ^ and CKD ^ † ^ care. The majority of participants (80.1%) indicated high levels of satisfaction with the system./ Level VI
Lew, et al., 2017 / Peritoneal Dialysis International/ United States	Transverse (descriptive)/ Home-based / PD ^ †† ^	Examine the use of remote biometric monitoring devices.	Remote biometric monitoring and audio and video communication by the Internet.	The RM ^ ‡ ^ is viable and capable of optimizing adherence to treatment and communication between the patient and the clinical team./ Level VI
Ishani, et al., 2016/ American Journal of Kidney Diseases/ United States	RCT * Home-based / GFR ^ || ^ < 60 mL/min/1.73 m ^2^, non-dialysis	Evaluate the feasibility and impact on health outcomes of the interprofessional telehealth program.	LifeView device; AmericanTeleCare, with tools (blood pressure cuff, scale, glucometer, pulse oximeter, stethoscope, and web camera) and access to the clinical team.	There was no difference between the groups for any component of the primary outcome: all-cause mortality, hospitalization, emergency department visits, or ward admission./ Level II
Migliozzi, et al., 2015/ Blood Pressure Control/ United States	Prospective cohort/ Home-based / Kidney transplant patients	Describe the RM ^ ‡ ^ blood pressure and drug management program.	RM ^ ‡ ^ of blood pressure with pharmacist management of drug therapy.	Significant reductions in mean systolic and diastolic were observed at 30, 90, 180, and 360 days after initiation of the program (p <0.05)./ Level IV
Rifkin, et al., 2013 / Blood Pressure Monitoring/ United States	RCT * Home-based/ Elderly hypertensive patients, CKD ^ † ^ stage 3.	To evaluate the applicability of RM ^ ‡ ^ to blood pressure.	Automatic blood pressure measurement device with data transmission to the health center.	Both groups obtained a reduction in systolic levels, without statistical significance. The IG ^ § ^ showed greater adherence to pressure checks./ Level II
Berman, et al., 2011/ Telemedicine and e-Health/ Hawaii	RCT * Home-based/ HD ^ ¶ ^	To determine whether home-based interventions using telehealth can optimize health outcomes and be economically sustainable.	Commercial Home Monitoring (VitelCare Turtle 500).	Nurse-supervised RM ^ ‡ ^ can improve health outcomes, with cost reductions, hospitalizations, and emergency room care./ Level II
**2. Teleconsultation**				
Cheung, et al., 2020 / Journal of Palliative Medicine / United States	Transverse / Hemodialysis clinics/ HD ^ ¶ ^	To determine the feasibility and acceptability of telepalliative care in rural dialysis unit.	Teleconsultation of Nursing in palliative care.	More than 80% of the participants reported that the teleconsultation was as good as the face-to-face one, and 41% found it better. In addition, 81% of the patients declared the teleconsultation relevant, 58% reported new learning about their condition and 27% revealed changes in their perception of dialysis./ Level VI
Kazawa, et al., 2020 / BMC Nursing/ Japan	RCT ^ * ^ Home-based/ Diabetics CKD ^ † ^	To examine the effectiveness between nursing teleconsultations and face-to-face care in promoting behavioral changes.	Nursing teleconsultation and telephone follow-up.	Both groups showed similar behavioral changes. The IG ^ § ^ showed a better understanding of the severity of their disease, the need for self-care, and trust in nurses. The GC ** showed a higher degree of behavioral change about self-monitoring./ Level II
Kaier, et al., 2017 / Health Economics Review/ Germany	RCT *Home-based/ Post-transplanted living donor kidneys	To analyze the costs and savings of the telemedicine case management program after kidney transplantation.	Post-renal transplant follow-up with case management by teleconference.	Participan assisted by telemedicine had lower costs and hospitalizations. The mean difference in costs was € 4,945.07 *per* patient, p <0.001. The economy was favorable when applied to 15 patients./ Level II
Alazab, et al., 2016 / Rural and Remote Health/ Jordan	Quasi-experimental/ Home-based (rural areas)/ Pre-dialysis patients	To evaluate the impact of telenephrology on diagnosis, disease management, quality of life, time savings, and costs.	Nephrology Teleconsultation.	The lowest costs and waiting time with telehealth were verified, impacting the improvement of QoL ^ ‡‡ ^ ./ Level III
Gallar, et al., 2007 / Journal of Telemedicine and Telecare/ Spain	RCT ^ * ^ Home-based/ PD ^ †† ^ Home-based	To evaluate the use of telemedicine in the long-term management of stable patients with PD ^ †† ^ .	Nursing teleconsultation with a review of technique, catheter care, early detection, and prevention of peritonitis.	Telemedicine appears to be clinically useful in the long-term follow-up of PD ^ †† ^ patients, with encouraging costs and savings./ Level II
Prado, et al., 2006/ International Journal of Medical Informatics/ Spain	Transverse/ Clinics/ HD ^ ¶ ^	To present the feasibility of a personalized telehealth system in Nephrology (NEFROTEL).	Telehealth systems composed of Remote Access Units and Public Switched Telephone Networks.	The system was able to provide physiological knowledge, integrated and adapted to each patient. Demonstrated reliability in human motion monitor impact detection./ Level VI
Kariyawasam, 2005/ EDTNA/ERCA Journal of Renal Care / United Kingdom	Prospective cohort/ Ambulatory/ HD ^ ¶ ^ with hyperphosphatemia	To verify the effectiveness of telemedicine in helping to control phosphorus levels and reduce consultation times with nutritionists.	Telemedicine unit with follow-up via nutritional teleconsultation.	There was a statistically significant reduction in phosphorus levels at 1, 3, and 6 months after nutritional counseling. Travel time was reduced and patients had the benefit of receiving the dietary information soon after the increased phosphate result. / Level IV
Michel, et al., 2000/ Journal of Telemedicine and Telecare/ France	Prospective cohort/ Clinics/ HD ^ ¶ ^	To compare the quality of care between face-to-face and remote care.	Follow-up by telehealth, with monthly and quarterly face-to-face consultations.	There was no difference in survival rate, number of deaths, and transplants between the groups. The evaluation for transplantation was lower (p<0.042) in IG ^ § ^ ./ Level IV
**3. Digital platform**				
Easom, et al., 2020 / Clinical Kidney Journal/ United States	RCT * Ambulatory/ CKD ^ † ^ stage 4 and 5 with ^ || ^ GFRof 30 mL/min/1.73 m ^2^	To evaluate the effectiveness of pre-dialysis education between the tele-education program and face-to-face care.	Online education platforms and face-to-face educational programs.	Reduced reports of lack of knowledge about RRT ^ |||| ^ after orientation sessions in both groups. Home modalities of RRT ^ |||| ^ were the favorites after the reorientations in both groups./ Level II
Cabacungan, et al., 2019 / Transplantation Proceedings/ United States	Transverse / Domiciliar/ Kidney donors and recipients	To test the usability and satisfaction of the app Talking About Live Kidney Donation Social Worker Intervention (TALK SWI).	Website and app with educational materials and behavioral intervention by social worker.	Most participants easily completed the technology tasks and preferred the educational app over traditional materials. There was high satisfaction with the intervention of counseling by a social worker./ Level VI
Weinhandl, et al., 2018 / Hemodialysis International/ United States	Prospective cohort/ Home-based / Home-based HD ^ ¶ ^	To evaluate the association between the telehealth platform (Nx2me *)* and the risk of changes during HD ^ ¶ ^ .	Telehealth Platform (Nx2me Connected Health).	IG ^ § ^ was associated with a lower risk of alterations due to all causes studied./ Level IV
Kiberd, et al., 2018 / Canadian Journal of Kidney Health and Disease/ Canada	Transverse/ Home-based / HHD ^ ¶¶ ^ and DP ^ †† ^	To determine the efficiency of an eHealth portal in optimizing the patient experience in home dialysis care.	Portal online eHealth (McKesson Canada, RelayHealth ^®^): sending text messages between patients and professionals, viewing message history, and accessing electronic medical records.	There were no differences in QoL ^ ‡‡ ^ and communication between the patient and the care team. Only 12 users answered the satisfaction questionnaire. Average monthly phone use decreased from 12.5 to 10 minutes after adopting the portal.
Barahimi, et al., 2017 / Iranian Journal of Kidney Diseases/ Iran	CT *** not randomized/ Ambulatory/ CKD ^ † ^ GFR ^ || ^ < 60 mL/min/1.73 m ^2^	To determine the effectiveness of virtual training in the impact of self-care.	Digital e-learning platform: http://barahimi.com/bmr.aspx	Both study groups showed statistically significant differences only in GFR ^ || ^ , with improvement in IG ^ § ^ ./ Level II
Gordon, et al., 2016 / Progress in Transplantation/ United States	Quasi-experimental/ Dialysis clinic / HD ^ ¶ ^ Hispanics	To evaluate the effectiveness of a website in promoting knowledge about kidney transplantation by living donors.	Website “Informate: Inform Yourself about Living Kidney Donation for Hispanics/Latinos”: treatment options; donation; benefits and risks; financial problems; immigrant issues; Cultural beliefs and myths.	There was a gain of knowledge in the two post-test tests, with statistical significance, especially in the sections on treatment and cultural beliefs./ Level III
Harrington, et al., 2014 / Blood Purification / United States	Transverse / Home-based / PD ^ †† ^ Outpatient Domiciliary	To examine the effectiveness of using a tablet app for asynchronous, real-time monitoring.	PD ^ †† ^ Remote aims to remind patients of sterile techniques and bag change procedures. It allows users to record vital signs, exchange data (% dextrose, volume infused and drained), and review medications and laboratory findings.	A total of 1,172 exchanges were recorded in 251 days. Compliance with the app ranged from 51 to 92%, with no major adverse events. The overall impression of the interface was 5.2 out of 10. Participants stressed the need to adjust the app to the level of patient experience and to simplify and automate data entry. Level VI
**4. Apps**				
Ong, et al., 2021 / Journal of the American Society of Nephrology/ Canada	RCT ^ * ^ Home-based / HD ^ ¶ ^ , PD ^ †† ^ and stage 3 to 5d	To compare the effectiveness of two digital apps in optimizing safety in drug therapy.	eKidneyCare app for medication management, blood pressure monitoring, symptom assessment, and laboratory testing screening for CKD ^ † ^ .	The eKidneyCare group had fewer total medication discrepancies compared to MyMedRec and also reduced the severity of clinically relevant medication discrepancies across all categories. Level II
Khoury, et al., 2020 / Journal of Medical Internet Research/ United Arab Emirates	Quasi-experimental/ Home-based / HD ^ ¶ ^	To estimate the effectiveness of dietary intervention with the smartphone app.	Diet diary app: Kidney Education for Lifestyle App (KELA.AE app).	There was an increase in average calorie intake and protein intake, with moderate to high effect size./ Level III
Ong, et al., 2016 / Journal of the American Society of Nephrology/ Canada	Quase-experimental/ Ambulatory/ CKD ^ † ^ stage 4 and 5	To determine the acceptability and clinical impact of the app for the self-care of patients with CKD ^ † ^ .	Mobile health app for smartphones.	There was 80% adherence to the app, with a statistically significant reduction in blood pressure. Report of more confidence in the control of clinical conditions./ Level III
Dey, et al., 2016 / SAGE Open Medicine/ United Kingdom	Quasi-experimental/ Home-based / PD ^ †† ^	To evaluate the acceptability of the technology by the patient and its effect on clinical interventions and QoL ^ ‡‡ ^ .	Interventions for self-management at home (Home PODs) with recognition of fluid problems, ability to change regimens, telephone dietary counseling, education via web resources, and access to clinical records.	There were no statistically significant differences between the start and end of follow-up. The high retention and satisfaction rates indicated high acceptability of the technology. Satisfaction was high, with no significant change in QoL ^ ‡‡ ^ score at the end of the program. The important characteristics of Home PODs for patients were ease of use, efficacy, and safety. Level III
Diamantidis, et al., 2015 / Clinical Journal of the American Society of Nephrology/ United States	Transverse/ Home-based/ GFR ^ || ^ < 60 mL/min/1.73 m ^2^	To examine the feasibility of the counseling mobile app on the safe use of medicines in CKD ^ † ^ .	Smartphone app for the consultation of medications with answers from the personal digital assistant, through images and alert text to emphasize the safety responses.	The high overall satisfaction with the app was verified, with emphasis on the digital assistant group compared to the short message service group. Only three errors were recorded among the 60 medication consultations./ Level VI
Doyle, et al., 2009/ Journal of Renal Care/ Ireland	Quasi-experimental/ Households/ CKD ^ † ^ stage 2 to 5	To develop and evaluate the app that stimulates the engagement of patien in the management of their clinical condition.	The smartphone app, MiKidney, with exercise tracker, daily record of exercises performed, reminder alerts, a notes section, a scoring scheme that provides feedback to users, and motivational messages.	There was a significant improvement in the walking test, total cholesterol, LDL ^ ††† ^ , waist circumference, and body fat. The MiKidney app was considered easy to navigate and 3/4 of the participants felt comfortable with the technology./ Level III
**5. Multimodalities**				
Polanco, et al., 2021 / Therapeutic Apheresis and Dialysis/ Dominican Republic	Longitudinal transverse/ Dialysis clinic/ PD ^ †† ^ manual and automated.	Report on the telemedicine protocol of the PD ^ †† ^ program in the context of the COVID-19 pandemic.	Multi-professional teleconsultation by video calls, photos, and text messages.	There were no statistical differences in the rates of peritonitis, hospitalization, and transfer to HD ^ ¶ ^ compared to care absent from telemedicine./ Level IV
Amici, et al., 2021 / International Urology and Nephrology/ Italy	Quasi-experimental/ Home-based / Automated PD ^ †† ^	Determine the impact of telemedicine technology with RM ^ ‡ ^ compared to traditional technology on organizational, social, and economic aspects.	Automated PD ^ †† ^ RM ^ ‡ ^ and telephone follow-up.	There was early detection of clinical problems, reduction of unscheduled visits, hospitalization, and telephone contact between patients and caregivers with the clinic. The RM ^ ‡ ^ system led to the relevant savings of € 335 (average *per* patient/month)./ Level III
Bunch, et al., 2020 / Blood Purification / Colombia	Prospective cohort / Clinics / PD ^ †† ^	Describe the organization of care for PD ^ †† ^ patients in the face of the COVID-19 pandemic and the impact on adherence to treatment and other health outcomes.	Implementation of telehealth with teleconsultation, videos, text messages, and teletriage.	There were no statistically significant differences in peritonitis rates. There was an increase in interactions between the patient and the clinic, with a decrease in evaluations of the catheter exit site./ Level IV
Milan, et al., 2019 / Nephron Clinical Practice/ Italy	Transverse/ Home-based/ Automated PD ^ †† ^	Evaluate the usefulness of the automated ^ †† ^ PD RM ^ ‡ ^ system.	Automated PD ^ †† ^ RM ^ ‡ ^ and telephone follow-up.	The number of night alarms, visits to the clinic, time spent and distances traveled were significantly lower in IG ^ § ^ . Patients reported ease of use of the RM ^ ‡ ^ system, and satisfaction with the level of interaction with the team and the timely resolution of technical problems. The intervention proved to be cost-effective./ Level VI
Kelly, et al., 2019 / BMJ Open / Australia	RCT * Ambulatory/ CKD ^ † ^ stage 3 to 4	To evaluate the feasibility and acceptability of telehealth intervention in dietary self-management in CKD ^ † ^ .	RM ^ ‡ ^ by phone calls and text messages.	The program showed high acceptability and usability among the groups, with 96% of the interventions completed. There was satisfaction with the frequency of contacts and the model was seen as an acceptable and personalized alternative to face-to-face clinical consultations./ Level II
Warner, et al., 2018 / JMIR Cardio/ United Kingdom	Transverse/ Home-based / CKD ^ † ^	To evaluate the usability and acceptability of blood pressure telemonitoring technology.	Blood pressure monitor by bluetooth and smartphone app.	The usability of the monitoring system was high. Pressure variability was significant over 30 and 90 days, indicating that the greatest variation was short-term./ Level VI
Aberger, et al., 2014 / Telemedicine and e-Health/ Iceland	Transverse (descriptive)/ Home-based / Post-kidney transplant patients	To optimize blood pressure levels, involvement, and medication adherence.	Good Health Gateway digital portal and blood pressure check monitor (adherence feedback and pressure monitoring).	There was a statistically significant reduction in mean systolic and diastolic pressures of 6 mmHg and 3 mmHg, respectively./ Level VI
**6. Telephone contact**				
Fallahpour, et al., 2020 / Nursing and Midwifery Studies/ Iran	RCT * Home-based / People over 60 in HD ^ ¶ ^	To evaluate the effects of telephonic care on physiological and psychological stressors.	Telenursing by telephone monitoring, with face-to-face educational sessions.	The mean scores for physiological and psychosocial stressors were reduced in GI ^ § ^ after follow-up, with no changes in GC**. There were differences between the groups in terms of the mean post-test scores and the mean pre- and post-test differences for both stressors: physiological and psychosocial./ Level II
Shahsavani, et al., 2019 / Journal of Evolution of Medical and Dental Sciences/ Iran	Quasi-experimental / Home-based / HD ^ ¶ ^	To investigate the effect of telenursing on health promotion behaviors.	Telenursing care with follow-up via telephone contact.	The IG ^ § ^ showed statistically significant differences in the dimensions of exercise, stress, responsibility, and interpersonal relationships. There were no differences in the nutrition and spirituality dimensions. / Level III
Tan, et al., 2018 / AMJ Nephrology / United States	Prospective cohort/ Ambulatory/ CKD ^ † ^ stage 1 to 5	To determine whether adherence to visits and clinical outcomes in remote CKD ^ † ^ are comparable to conventional face-to-face care.	Telenephrology with follow-up via telephone calls.	Cancellation of appointments was reduced by half, with a higher frequency of attendance at telenephrology appointments. The incidence of death, CKD ^ † ^ dialysis, or creatinine doubling was similar in both groups./ Level IV
Cao, et al., 2018 / Journal of Clinical Nursing/ China	CT ^ *** ^ not randomized/ Home-based/ PD ^ †† ^	Investigating the effectiveness of instant messaging apps in monitoring PD ^ †† ^ patients.	Usual caution along with sending text messages.	IG ^ § ^ had higher levels of serum albumin and hemoglobin, and lower levels of phosphorus, calcium-phosphorus product. There was a better level of satisfaction, with statistical significance, in all these variables. / Level II
Hung, et al., 2018 / Journal of Medical Internet Research/ China	Retrospective cohort/ Home-based / CKD ^ † ^ non-dialytic, dialytic and absence of CKD ^ † ^	To evaluate the relationship between telehealth program adherence rates and hospitalizations in patien with and without CKD ^ † ^ .	Telehealth program of telephone follow-up with the monitoring of biometric data (electrocardiography, blood pressure, heart rate, and pulse oximetry).	The compliance rate had a three-phase relationship with cardiovascular and all-cause hospitalizations. Low or very high compliance rates were associated with a higher risk of hospitalization. Patients with CKD ^ † ^ were associated with a higher risk of hospitalization, and dialysis patients had an increased risk when they had low adherence rates, compared to patients with normal kidney function or non-dialysis CKD ^ † ^ . / Level IV
Gross. et al., 2017 / Contemporary Clinical Trials/ United States	RCT ^ * ^ Home-based / Patients awaiting kidney transplantation	To test the effectiveness and establish the feasibility of stress reduction based on mindfulness adapted by telephone.	Stress reduction program based on Mindfulness Stress Reduction (MBSR) by meditation and collective Yoga with the community classroom by telephone.	There were low changes in the level of anxiety, which did not differ during follow-up. Mental QoL ^ ‡‡ ^ in IG ^ § ^ improved significantly and 90% of participants reported that mindfulness practice helps manage stress./ Level II
Poorgholami, et al., 2015 / International Journal of Community Based Nursing & Midwifery/ Iran	RCT ^ * ^ Home-based / HD ^ ¶ ^	To examine the effects of telephone monitoring on the level of hope in a self-care education program.	Telephone monitoring in an education program for self-care.	*A priori*, there were no significant differences between the groups for the hope scores. After the intervention, the level of hope in IG ^ § ^ was significantly higher, especially in those with telephone follow-up./ Level II

* RCT = Randomized Clinical Trial

† CKD = Chronic Kidney Disease

‡ RM = Remote Monitoring

§ IG = Intervention Group

|| GFR = Glomerular Filtration Rate

¶ HD = Hemodialysis

* CG = Control Group

†† PD = Peritoneal Dialysis

‡‡ QoL = Quality of Life

§§ SMS = Short Message Service

|||| RRT = Renal Replacement Therapy

¶¶ HHD = Home Hemodialysis

*** CT = Clinical Trial

††† LDL = Low Density Lipoprotein

### Characterization of the included studies

The final sample of publications covered 48 articles published between 2000 and 2021, with a predominance of the years 2020 (10/20.8%) ^(^ 15 ^-^ 24 ^)^, 2019 (7/14.6%) ^(^ 25 ^-^ 31 ^)^ and 2018 (6/12.5%) ^(^ 32 ^-^ 37 ^)^. That said, there were a total of 39 different journals, of which seven presented more than one publication on the subject, three with scope in telemedicine (Journal of Medical Internet Research, Telemedicine and e-Health and Journal of Telemedicine and Telecare) and four in nephrology (Peritoneal Dialysis International, Journal of the American Society of Nephrology, Journal of Nephrology and Blood Purification). 

Among the countries with the highest number of publications, the United States (USA) obtained greater prominence, with 35.4% (17) of the productions on the theme, followed by Iran with four publications, the other seventeen countries presented between one and three studies. The second continent with the highest number of scientific records was the European (13/27/%), Latin America was represented by three publications, from Colombia ^(^ 24 ^-^ 29 ^)^ and Dominican Republic ^(^ 38 ^)^. 

The characterization of the environment where the research was developed revealed that the home setting had the highest prevalence (36/75%), followed by dialysis clinics (6/12.5%) and outpatient clinics (6/12.5%). Individuals with CKD in conservative treatment (14/29.2%), peritoneal dialysis (13/27.1%), and hemodialysis (12/25%) stood out. Kidney transplant recipients were included in five studies and kidney donors/recipients comprised the sample of one study. The multidisciplinary team worked in fifteen (31.3%) studies, other professionals with greater presence were nurses (25%), followed by nutritionists (8.3%), pharmacists (8.3%), physicians (6.3%) and social workers (4.2%) the specialty of the health professional was not informed in eight (16.7%).

Regarding the designs of the studies identified in this integrative review, clinical trials, randomized or not, total 17 (35.4%), descriptive observational studies comprised 13 (27.1%) productions, cohort studies represented nine articles (18.8%) and quasi-experimental, of the pre and post type, constituted eight (16.7%) publications. It was found that 37.5% (18) of the articles presented level II of evidence, followed by level IV with 22.9% (11).

The outcomes reported showed intense diversity among the studies, ranging from clinical indicators (hospitalization, peritonitis, emergencies, mortality), laboratory parameters (hemoglobin, phosphorus, albumin, calcium, cholesterol), blood pressure levels, to variables related to user satisfaction. Overall, behavioral changes, quality of life, and usability of the technological tools applied also constituted the spectrum of weighted outcomes, with costs evaluated in only five studies.

It is noteworthy that no included study showed unfavorable results to telehealth interventions, although fifteen of them reported no statistically significant differences between the groups investigated. Therefore, favorable implications were indicated in 32 studies, whose outcomes analyzed were positively inclined to remote assistance with hypothesis tests presenting statistical significance (p<0.05) in 24 investigations.

### Remote monitoring devices

It included studies that worked with electronic devices capable of capturing biometric data of the user and sending it to the responsible team, which may be of immediate online transmission or not. Among them, the use of a wristwatch ^(^ 21 ^)^, medication tray ^(^ 17 ^)^, PD machine ^(^ 22 ^,^ 29 ^)^ and a monitoring station with a camera, microphone and monitor ^(^ 31 ^)^ were identified. 

As for the articles arranged in this category, there were a total of 13 (27.1%), so all were developed in a home environment. Moreover, it is noteworthy that the predominance of the target population occurred from patients on peritoneal dialysis ^(^ 22 ^,^ 29 ^,^ 31 ^,^ 39 ^-^ 40 ^)^, followed by individuals undergoing conservative treatment ^(^ 21 ^,^ 30 ^-^ 42 ^)^, transplanted patients ^(^ 17 ^,^ 43 ^)^ and hemodialysis ^(^ 18 ^,^ 44 ^)^. 

The USA was the site of the development of eight studies ^(^ 17 ^-^ 18 ^,^ 30 ^,^ 39 ^-^ 43 ^)^, while the others occurred in Hawaii ^(^ 44 ^)^, Taiwan ^(^ 21 ^)^, Colombia ^(^ 29 ^)^, and Italy ^(^ 22 ^,^ 31 ^)^. Regarding the year of publication, it was found that seven of them occurred in 2019 ^(^ 29 ^,^ 31 ^)^ and 2020 ^(^ 15 ^-^ 16 ^,^ 21 ^-^ 22 ^)^, the others between 2011 and 2017, so six were designed as clinical trials ^(^ 17 ^-^ 18 ^,^ 21 ^,^ 41 ^-^ 42 ^,^ 44 ^)^, and the other seven as observational studies. 

The multidisciplinary team was the category with the greatest performance in the strategy, conducting five investigations ^(^ 22 ^,^ 30 ^-^ 31 ^,^ 41 ^-^ 42 ^)^. Nurses led four studies ^(^ 29 ^,^ 39 ^-^ 40 ^,^ 44 ^)^, followed by pharmacist ^(^ 43 ^)^ and nutritionist ^(^ 18 ^)^. 

Finally, it is noted that, about the resul of the category under analysis, all studies showed positive trends with the app of remote support: decrease in hospitalizations ^(^ 18 ^,^ 22 ^,^ 29 ^,^ 44 ^)^, increase in levels of self-care ^(^ 21 ^,^ 30 ^,^ 39 ^-^ 40 ^)^, reduction in the use of medications ^(^ 17 ^,^ 30 ^)^, decrease in blood pressure levels ^(^ 41 ^,^ 43 ^)^, emergency costs ^(^ 44 ^)^, decrease in negative perception with dialysis treatment ^(^ 40 ^)^ and decrease in emergency care ^(^ 18 ^,^ 42 ^,^ 44 ^)^. 

### Teleconsultation

The teleconsultation category included studies that used videoconferencing as a service methodology, similarly called teleconference or video call. Its application occurred predominantly in the home environment ^(^ 16 ^,^ 45 ^-^ 47 ^)^ and in clinics ^(^ 19 ^,^ 48 ^-^ 49 ^)^, with the outpatient setting as the focus in only one study ^(^ 50 ^)^. The target population of this strategy was individuals on hemodialysis who were part of four study treatments ^(^ 16 ^,^ 46 ^)^, PD ^(^ 47 ^)^ and transplant recipients ^(^ 45 ^)^. 

The teleconference represented eight studies, with a frequency of 16.7% of the total, carried out in the USA ^(^ 19 ^)^, Germany ^(^ 45 ^)^, France ^(^ 48 ^)^, Jordan ^(^ 46 ^)^, Spain ^(^ 47 ^,^ 49 ^)^, the United Kingdom ^(^ 50 ^)^ and Japan ^(^ 16 ^)^. Most of the articles were published between 2000 and 2007 ^(^ 47 ^-^ 50 ^)^, with the other two studies dating from 2016 ^(^ 46 ^)^ and 2017 ^(^ 45 ^)^, and only in 2020 two other studies ^(^ 16 ^,^ 19 ^)^. The clinical trial was used as a method in three studies ^(^ 16 ^,^ 45 ^,^ 47 ^)^, while the cohort and cross-sectional designs were verified in four studies ^(^ 19 ^,^ 48 ^-^ 50 ^)^. 

The professionals with the greatest performance in this strategy were physicians ^(^ 46 ^,^ 48 ^)^ and nurses ^(^ 16 ^,^ 47 ^)^ who worked in two studies each, together with the nutritionist ^(^ 50 ^)^. However, two publications did not specify which professional performed the care ^(^ 44 ^,^ 49 ^)^. 

As a result, teleconsultation was characterized as better or equally effective as face-to-face consultation by 80% of the participan, with the development of new skills and better perception of the therapy ^(^ 19 ^)^. There was a positive impact on cost reduction ^(^ 45 ^-^ 47 ^)^, waiting for time for care ^(^ 46 ^)^ and nutritional control of phosphorus levels ^(^ 50 ^)^. Although teleconsultation did not present statistical superiority with renal transplant patients, there were no differences regarding the survival rate in this population ^(^ 48 ^)^. 

### Digital platform

Digital platforms were characterized by all those studies that developed, tested, or applied patient care strategies through websites or online platforms hosted in the World Wide Web environment. Seven (14.6%) studies were classified from the USA ^(^ 20 ^,^ 25 ^,^ 36 ^,^ 51 ^-^ 52 ^)^, Canada ^(^ 34 ^)^, and Iran ^(^ 53 ^)^, published between 2014 and 2020, except for 2015. There was a predominance of cross-sectional studies ^(^ 25 ^,^ 34 ^,^ 52 ^)^ and clinical trials ^(^ 20 ^,^ 53 ^)^, representing five studies, followed by a quasi-experimental design ^(^ 51 ^)^ and cohort ^(^ 36 ^)^. 

The population included all renal groups, expressed by: transplanted ^(^ 25 ^)^, hemodialysis ^(^ 36 ^,^ 51 ^)^, peritoneal dialysis ^(^ 52 ^)^, conservative ^(^ 53 ^)^, and mixed population ^(^ 34 ^)^ which included individuals on conservative and dialysis treatment. The home environment was the setting for four studies ^(^ 25 ^,^ 34 ^,^ 36 ^,^ 52 ^)^, while two used the outpatient setting ^(^ 20 ^,^ 53 ^)^ and one was directed to the clinics ^(^ 51 ^)^. Moreover, three productions did not identify the professionals working in the research ^(^ 34 ^,^ 36 ^,^ 51 ^)^, and the other studies were developed by a social worker ^(^ 25 ^)^, a multidisciplinary team ^(^ 52 ^)^ and a nurse, representing two productions ^(^ 20 ^,^ 53 ^)^. 

It was found that the use of the online platform can improve knowledge about dialysis therapies among users ^(^ 20 ^)^ and reduce the risk of complications in home hemodialysis ^(^ 36 ^)^. In consonance, the use of a self-care support portal for home dialysis provided a reduction in the need to contact the clinic ^(^ 34 ^)^, greater confidence and knowledge in performing the procedures in PD ^(^ 52 ^)^ and an improvement in glomerular filtration rates in those undergoing conservative treatment ^(^ 53 ^)^. 

### Apps

The strategy in question was characterized by using apps directed to certain electronic devices, such as cell phones or tablets. Its use was predominantly in the home setting, to cover seven studies ^(^ 15 ^,^ 32 ^,^ 54 ^-^ 58 ^)^. Parallel to the above, only one publication mentioned the outpatient setting ^(^ 58 ^)^ in this category. Among the population present in this category were individuals under conservative treatment, PD and HD, expressed, respectively, in three, two, and one study. 

Seven (14.6%) studies were identified that used apps as a remote service strategy, from the USA ^(^ 55 ^)^, Ireland ^(^ 57 ^)^, the United Kingdom ^(^ 56 ^)^, China ^(^ 32 ^)^, the United Arab Emirates ^(^ 15 ^)^ and Canada ^(^ 54 ^,^ 58 ^)^. In the first instance, a pilot study was presented in 2009, which evaluated the impact of an app regarding the expansion of knowledge of renal patients ^(^ 57 ^)^. In the second instance, there are three more studies carried out in 2015 ^(^ 55 ^)^ and 2016 ^(^ 56 ^,^ 58 ^)^, followed by the years 2020 ^(^ 15 ^)^ and 2021 ^(^ 54 ^)^. 

The quasi-experimental method was verified in four studies ^(^ 15 ^,^ 56 ^-^ 58 ^)^, the CT method was verified in two studies ^(^ 32 ^,^ 54 ^)^ and the cross-sectional method ^(^ 55 ^)^ was verified in one. Among these studies, the pharmacist was responsible for conducting two ^(^ 54 ^,^ 56 ^)^, the nutritionist for one ^(^ 15 ^)^ and the team for three others ^(^ 32 ^,^ 57 ^-^ 58 ^)^. 

Regarding the results, it is noted that the apps can increase the safety in the use of medications ^(^ 54 ^)^ and improve the intake of calories and proteins in patients under HD ^(^ 15 ^)^. In addition to what has been discussed, there is an improvement in albumin, hemoglobin and calcium-phosphorus levels in individuals under PD accompanied by an instant messaging device ^(^ 32 ^)^. It is also noteworthy that those undergoing conservative treatments also benefited from the promotion of self-care with a reduction of blood pressure levels ^(^ 58 ^)^, along with the verification of high rates of acceptability and satisfaction with the use of apps ^(^ 55 ^,^ 57 ^,^ 59 ^)^. 

### Multimodality

The multimodality category frames the studies that used, with the same relevance, diversified and joint TH strategies. As an example, there are teleconsultation and text messages ^(^ 24 ^,^ 26 ^,^ 38 ^)^, PD monitoring with telephone contact ^(^ 28 ^,^ 60 ^)^, blood pressure monitoring with app ^(^ 35 ^)^ and blood pressure monitoring with an online platform for data collection ^(^ 61 ^)^. Regarding the scenarios adopted, the home environment is presented in four studies ^(^ 28 ^,^ 35 ^,^ 60 ^-^ 61 ^)^, the dialysis clinics in two ^(^ 24 ^,^ 38 ^)^ and the outpatient clinic in one ^(^ 26 ^)^. 

Among the sample population present in this category, PD patients stand out, which comprise four studies ^(^ 28 ^,^ 38 ^,^ 60 ^,^ 62 ^)^. In the others, there were transplanted individuals ^(^ 61 ^)^ and under conservative treatment ^(^ 26 ^,^ 35 ^)^, with the exclusion of patients on HD. The strategy under analysis obtained an overall calculation of seven articles (14.6%), of which two ^(^ 28 ^,^ 60 ^)^ present Italy as the country of origin. The others came from different countries in Latin America ^(^ 24 ^,^ 38 ^)^, Europe ^(^ 35 ^,^ 61 ^)^ and Oceania ^(^ 26 ^)^, however, none of them were from North America. 

Regarding the year of publication, it was found that the pandemic period was prevalent, between 2019 and 2021 ^(^ 24 ^,^ 26 ^,^ 28 ^,^ 38 ^,^ 60 ^)^, to account for five of them, the others being published in 2018 ^(^ 35 ^)^ and 2014 ^(^ 61 ^)^. Moreover, the multimodalities presented the cross-sectional design as predominant, with four articles ^(^ 28 ^,^ 35 ^,^ 38 ^,^ 61 ^)^; the others used cohort ^(^ 24 ^)^, quasi-experimental ^(^ 60 ^)^, and CT ^(^ 26 ^)^ methods. Regarding the guardians, four ^(^ 24 ^,^ 8 ^,^ 35 ^,^ 38 ^,^^)^ were composed of a multidisciplinary team and, in the others, the pharmacist ^(^ 61 ^)^ and nutritionist ^(^ 26 ^)^. 

According to the results presented, it was understood that telephone follow-up and text messages were acceptable and feasible ^(^ 26 ^,^ 28 ^)^, and there was a statistically significant reduction in the rates of peritonitis in a study that associated teleconsultation, telescreening and text messages ^(^ 24 ^)^, as well as in the reduction of blood pressure when monitored remotely ^(^ 64 ^)^. Nevertheless, in a study conducted during the 2020 pandemic, the indicators of peritonitis and hospitalizations did not present statistical differences when compared to patients who did not participate in TH ^(^ 38 ^)^. 

### Telephone contact

This category refers to studies that applied only telephone contact as a form of remote monitoring, that is, videos or images were not applied. Thus, there were six studies (12.5%), five of them involved the home environment ^(^ 23 ^,^ 27 ^,^ 33 ^,^ 64 ^-^ 65 ^)^, and another the outpatient setting ^(^ 37 ^)^. Iran ^(^ 23 ^,^ 27 ^,^ 64 ^)^, the USA ^(^ 37 ^,^ 65 ^)^ and China ^(^ 33 ^)^ were the countries that published the most in this modality. It should be noted that no developing country was included in it. 

Regarding the year of publication, it was verified that, except for 2016, all studies were published between 2015 and 2020, since in 2018 there were two publications ^(^ 33 ^,^ 37 ^)^. Regarding populations, it was found that patients on HD were in three studies ^(^ 23 ^,^ 27 ^,^ 64 ^)^, while conservative treatment was only in one ^(^ 35 ^)^, and the others addressed a mixed population ^(^ 33 ^,^ 65 ^)^. 

Telephone contact was the strategy of three studies developed as clinical trials ^(^ 23 ^,^ 64 ^-^ 65 ^)^, two cohorts ^(^ 33 ^,^ 37 ^)^ and one quasi-experimental study ^(^ 27 ^)^. The professional nurse was in four studies ^(^ 23 ^,^ 27 ^,^ 33 ^,^ 64 ^)^ with the use of telephone, the team in only one ^(^ 65 ^)^, as well as the medical professional ^(^ 37 ^)^. 

The reported results indicated that HD patients under telephone monitoring presented lower physiological and psychosocial stressors ^(^ 23 ^)^. They also showed that telephone contact by nurses provided the best results regarding physical exercise, stress, and responsibility ^(^ 27 ^)^, so this method of care enabled greater adherence to consultations among individuals who lived far from clinical centers, with increased frequency and reduced cancellations ^(^ 37 ^)^. In addition to the above, it was noted that the higher risk of hospitalization was verified among those with dialysis CKD when they did not participate in telephone monitoring ^(^ 33 ^)^, and, ultimately, stress reduction was observed in renal transplant patien who practiced mindfulness by telephone ^(^ 65 ^)^. 

## Discussion

Technological development has allowed the diffusion of different social interaction tools that can be applied in the health area, which is denoted by the various strategies found in the studies included in this review, grouped by similarity into six categories in which remote monitoring devices were the most frequent. The investigation supported by the American Society of Nephrology pointed out possibilities of interaction between patients and caregivers, intermediated by digital resources ^(^ 6 ^)^, as in another survey that identified a wide diversity of these resources: virtual consultations, text messages, sending images via online questionnaires, optimized use of smartphones, among others ^(^ 8 ^)^. 

In addition to offering numerous possibilities, telehealth proved to be transversal and usable by the Nephrology team, although with greater applicability by individual professionals, such as nurses, pharmacists, nutritionists, and physicians. Although the area has historically been linked to devices and technological devices – dialysis machines –, the use of telehealth as a means of patient care from the resources already available gained visibility during the pandemic, when professionals and services had to adapt to social distancing ^(^ 8 ^,^ 10 ^)^. 

Regarding the places of publication, the predominance of studies from the United States and Europe evidences the potential interest, in these places, in the development of telehealth in Nephrology and the incorporation of information technology, mediating health care. In Latin America, there have been few publications restricted to remote monitoring in PD, although the published evaluation of telehealth in different regions of the globe has indicated an exponential increase in telephone consultations in Brazil, Ecuador, and Peru ^(^ 9 ^)^. Moreover, it also indicates that in this region the use of telehealth is extremely low and is linked to the scarce financial resources, prejudice and resistance of health professionals. 

However, the adequacy of services in the face of the incorporation of telehealth is inevitable and has gained greater evidence and robustness in the COVID-19 pandemic demonstrated by the high number of articles published between 2019 and 2021, twenty in total. Similar results were found in another survey that, in the same way, verified the exponential and accelerated use of telehealth in all areas of health in this period ^(^ 8 ^)^. In Nephrology, the pandemic in a certain way forced its advancement and implementation, so that more discussions about the barriers and difficulties arose, while its benefits, although mostly intuitive, were confirmed ^(^ 5 ^)^. 

In this sense, it was remarkable the remote monitoring via electronic devices, telephone contact, and teleconsultation performed by doctors and nurses with people in HD and PD in the home environment, since twelve studies were thus characterized. In Singapore, the implementation of telehealth during the pandemic was essentially via teleconsultation of the doctor and nurse ^(^ 10 ^)^. The use of smartphones and the availability of free videoconferencing platforms reinforced the practice of teleconsultation and remote monitoring of patients, demonstrating to be economically viable due to the reduction of visits to the treatment center, hospitalizations, and emergency care ^(^ 8 ^)^. 

This result reinforces the use of telehealth in nephrology, especially for the remote monitoring of people who perform dialysis therapies at home. The complexity of the therapy and its risks demand from the patient a greater capacity for self-care and interaction with the health team, which is allowed by the use of communication technologies ^(^ 5 ^)^. Moreover, a potential benefit of telehealth in Nephrology is the greater acceptance of home therapies where, despite the distance, access to professionals by the patient would be optimized ^(^ 4 ^)^. 

Finally, the results reported in the studies did not indicate the inferiority of telehealth compared to traditional care or non-acceptance, despite the different outcomes analyzed and the strategies used. In this sense, telehealth still lacks robust evidence about its clinical and economic effectiveness, acceptability, and viability from the perspective of the service, professional, and patient ^(^ 5 ^)^. 

It was also found that despite the accelerated use of telehealth designed by the pandemic, the theme has been the object of research in the field of nephrology for more than 20 years, proving to be feasible from the point of view of the user and provider. In addition, with the multiplicity of technological tools used in the care of CKD, it is perceived that guidelines are necessary to guide professional performance and the development of research that can unveil the effectiveness of technologies and improve access to them, to resolve regional inequities. In addition, it was found that there are few and restricted studies involving renal transplant patients whose monitoring is outpatient and who, due to exposure to the risks of immunosuppression and polypharmacy, could greatly benefit from telehealth.

In this review, the comparability on the effectiveness between the various tools was not possible due to the diversity of methodological designs in the included studies, as well as the population studied and the outcomes evaluated by the study, the particularities pointed out in other publications ^(^ 3 ^-^ 4 ^)^. As limitations, we can mention the possibility of selection bias, which was controlled by the number of reviewers who selected the articles independently, the impossibility of access to the full text of four articles after exhausting the attempts, the absence of some data on the professional involved in the research, which did not prevent the possibility of characterization of the evidence listed. 

## Conclusion

The telehealth strategies used in the care of people with CKD have several forms and implementations identified in this study: remote monitoring devices, teleconsultation, digital platforms, apps, telephone contacts and strategies that associate two or more possibilities of telehealth, being feasible for the renal population in any phase of the disease and applicable by different health professionals with emphasis on the home environment. The application of these means of assistance has been going on for more than a decade and has gained emphasis with the restrictive measures of movement imposed by the COVID-19 pandemic. Telehealth care for people with CKD has been shown to reduce costs and improve clinical, laboratory and behavioral outcomes in patients, especially on dialysis. In addition, no study presented outcomes with a lower impact related to the use of telehealth strategies.

This review pointed out an important research gap involving developing countries whose inequity of access, high indirect costs with dialysis treatment for access to more distant centers, and emergency care could be mitigated with the implementation of remote care by the care team. Similarly, the population of renal transplant recipients has been explored with telehealth in few and restricted publications. However, this study that analyzed the evidence in the literature showed that several first-world countries have already advanced towards the incorporation of digital technologies, expanding the possibilities of providing effective care to the renal population by devices of common use and accessible to most people.

Despite the existing barriers to the incorporation of these practices in health, the articulation of technologies available to users, such as smartphones, should be widely explored by health services to expand access to treatments and specialized professionals, as well as to promote quality of life through increased knowledge and monitoring, with consequent reduction of health risks and disease control.
